# Feasibility of sun and magnetic compass mechanisms in avian long-distance migration

**DOI:** 10.1186/s40462-018-0126-4

**Published:** 2018-06-06

**Authors:** Rachel Muheim, Heiko Schmaljohann, Thomas Alerstam

**Affiliations:** 1 Department of Biology, Lund University, Biology Building B, 223 62 Lund, Sweden; 2 Institute for Biology und Environmental Sciences (IBU), Carl von Ossietzky University of Oldenburg, Carl-von-Ossietzky-Straße 9-11, D-26129 Oldenburg, Germany; 3 Institute of Avian Research, Vogelwarte Helgoland, An der Vogelwarte 21, 26386 Wilhelmshaven, Germany; 4 Department of Biology, Lund University, Ecology Building, 223 62 Lund, Sweden

**Keywords:** Sun compass, Magnetic compass, Bird migration, Orientation

## Abstract

Birds use different compass mechanisms based on celestial (stars, sun, skylight polarization pattern) and geomagnetic cues for orientation. Yet, much remains to be understood how birds actually use these compass mechanisms on their long-distance migratory journeys. Here, we assess in more detail the consequences of using different sun and magnetic compass mechanisms for the resulting bird migration routes during both autumn and spring migration. First, we calculated predicted flight routes to determine which of the compasses mechanisms lead to realistic and feasible migration routes starting at different latitudes during autumn and spring migration. We then compared the adaptive values of the different compass mechanisms by calculating distance ratios in relation to the shortest possible trajectory for three populations of nocturnal passerine migrants: northern wheatear *Oenanthe oenanthe*, pied flycatcher *Ficedula hypoleuca*, and willow warbler *Phylloscopus trochilus*. Finally, we compared the predicted trajectories for different compass strategies with observed routes based on recent light-level geolocation tracking results for five individuals of northern wheatears migrating between Alaska and tropical Africa. We conclude that the feasibility of different compass routes varies greatly with latitude, migratory direction, migration season, and geographic location. Routes following a single compass course throughout the migratory journey are feasible for many bird populations, but the underlying compass mechanisms likely differ between populations. In many cases, however, the birds likely have to reorient once to a few times along the migration route and/or use map information to successfully reach their migratory destination.

**Electronic supplementary material:**

The online version of this article (10.1186/s40462-018-0126-4) contains supplementary material, which is available to authorized users.

## Background

It is well established that birds use a variety of orientation and navigation mechanisms to find their way during migration. Young, inexperienced birds on their first migration are generally assumed to use a genetically encoded program, providing them with information on the direction and distance to migrate [1–3]. Navigational map information collected during this first migration allows them then to navigate back to the known breeding area and during future migrations, as has been shown by several displacement experiments [4–6]. Birds use a variety of different compass mechanisms for orientation during migration, based on celestial or geomagnetic cues. Celestial compass cues (stars, sun, skylight polarization patterns) provide birds with directional information relative to a true geographic reference (e.g., geographic North) [7–12]. Magnetic compass information is based on the alignment of the Earth’s magnetic field, with magnetic North (or magnetic South) as reference [13, 14]. Because of irregularities and changing properties of the geomagnetic field, the magnetic poles do not coincide with the geographic poles [15]. This may pose problems for migratory birds using celestial and magnetic compass cues interchangeably along their migratory journey, because they are exposed to a changing relationship between the two reference systems, i.e., changing magnetic declination, which is the difference between magnetic and geographic North/South [16–19]. Birds have been shown to regularly calibrate the different compasses with each other [20–23], but there is an ongoing debate about which of the compass mechanisms acts as the primary reference, and how this compass information is translated into the migration trajectories that we observe in nature.

One possibility to shed light on this question is to compare predicted trajectories based on assumptions of constant orientation according to different compass mechanisms with the observed geometry of bird migration routes. A number of studies have predicted migration trajectories of birds for different celestial and geomagnetic compass mechanisms, usually by extrapolating known directional choices of passerine bird populations from orientation experiments or various tracking methods, and comparing the output with the known trajectories or goal areas of the respective populations (e.g. [18, 24–31]). Often, these studies did not find strong agreement with observed routes suggesting that the orientation task might be more complex than to simply follow a single compass course throughout the journey.

In this contribution we approach this question more systematically and in more detail than has previously been done to assess the consequences of using different compass mechanisms for the resulting bird migration routes for both autumn and spring migration. We focused primarily on migration routes of passerines and used three main approaches: First, we calculated predicted flight routes based on four types of sun compass and two types of magnetic compass mechanisms and discuss the geometric characteristics of these routes compared to great circle (orthodromes) and rhumbline routes (loxodromes). We discuss the suitability of routes only from a geometric point of view, disregarding geographic or ecological factors along the routes. We then compared the adaptive values of the different compass mechanisms by calculating distance ratios in relation to the shortest possible trajectory (the orthodrome, along the great circle) for three example populations of nocturnal passerine migrants, namely northern wheatears *Oenanthe oenanthe* migrating from Greenland to western Africa, and pied flycatchers *Ficedula hypoleuca* and willow warblers *Phylloscopus trochilus* migrating from northern Scandinavia to western and eastern Africa, respectively. Finally, we made a critical comparison between predicted trajectories for five compass strategies for autumn and spring migration and the observed routes based on recent light-level geolocation tracking results for five individuals of northern wheatears migrating between Alaska and eastern Africa [32]. These three lines of investigation allow us to draw novel conclusions about constraints in the feasibility of different compass mechanisms for long-distance migration depending on latitude and season, about the costs in terms of extra travel distance for different compass mechanisms, and about the likelihood for constant compass orientation on an intercontinental and global scale. We would like to stress that we only consider routes based on simple compass orientation which do not include pre-programmed directional changes requiring new start directions at specific locations along the migration route, as has been shown to occur in several bird populations (cf. [3, 33, 34]). Also, we do not include the possibility that birds may use map information to navigate to their migratory destination, despite of convincing evidence that migrants are able to compensate for displacements [4–6, 35–37], in some cases already during their first return migration during spring [6, 37].

## Simulations of bird migration routes based on different sun compass mechanisms

The first compass mechanism to be discovered and explored in birds was the time-compensated sun compass [7, 35]. Birds can determine the compass direction from the sun (or from sun-related cues like the skylight polarization pattern) by compensating, through their circadian clock sense, for the sun’s apparent daily movement in azimuth. This compass mechanism seems to be highly flexible, and differences in rates of sun azimuth changes during different hours of the day and at different latitudes and seasons may be taken into account [10, 11, 38–41]. Birds may use such a time-compensated sun compass also for orientation at the times of sunset (in the case of nocturnal migrants) or sunrise (in the case of diurnal migrants) [12, 42] (see section on time-compensated sunset compass below). Alternatively, birds could orient at a fixed angle relative to sunset or sunrise without time compensation (menotaxis) [43]; see section on fixed (menotactic) sunset compass below). Day migrants could theoretically take sun-compass readings once a day at noon or once an hour during the light hours of the day (see sections on time-compensated noon and time-compensated hourly sun compass below).

In this series of analyses, we simulated flight routes for unspecified model migrant populations based on different sun compass courses. We calculated the routes in daily steps of 200 km, determining a new course for each step based on astronomical conditions at each daily departure location/time, and assuming a constant geographic course within a step. In the case of the time-compensated hourly sun compass, which could be used by diurnal migrants, a new direction was determined once an hour (every 25 km) between 08:00 and 16:00. For simplicity, we assumed that the birds travelled each day, without making any stopovers along the journey. Thus, travel as well as migration speed were 200 km/d for the simulations, which is not unreasonable for long-distance migrants (see supplementary review table in [44]). We acknowledge, however, that daily travel speed can have a substantial influence on the resulting trajectories, as exemplified in Additional file 1: Figure S1. Autumn migration routes were simulated with 1 Sept as initial departure date, and spring migration routes with 1 April as departure date. These dates were chosen for generic model populations with no specific species in mind. Since the timing of the migration season affects sun compass routes, populations migrating earlier or later in the season will therefore migrate along slightly different routes (for example see Additional file 2: Figure S2). Autumn routes were simulated with initial departure directions of 90°, 135°, 180°, 225°, and 270°, from departure locations at latitudes 70°N, 50°N and 30°N. Spring migration routes were simulated with initial departure directions of 300°, 330°, 360°, 30°, and 60°, from departure locations at latitudes 30°S, Equator (0°) and 30°N. Unlike routes based on magnetic compass courses which are dependent on geographic latitude and longitude (see below), sun compass routes are only dependent on latitude, but not longitude. The simulations were carried out in MATLAB R2008a-R2016b (The MathWorks Inc., Natick, MA, USA). Sun positions were calculated using the Matlab script sun_positionR.m by Vincent Roy based on the solar position algorithm by [45].

### Time-compensated sunset compass

#### Assumptions

Birds are assumed to use their time-compensated sun compass to establish their orientation around sunset [12, 42, 46]. We focus on sunset here, since a large proportion of passerines migrate at night and are believed to establish their departure direction around sunset [12, 22, 43, 47]. For diurnal migrants, a time-compensated sunrise compass would work equally well. This compass will allow migrants to compensate for the change in sun azimuth during the hours before and after sunset according to the local conditions at the departure site. The rate of change of sun azimuth at sunset depends on latitude, as explained by Alerstam and Pettersson [48]. When the birds establish their orientation at a new site without having reset their inherent circadian clock to local time at the new site (still having their daily clock in phase with the time at their initial or former departure site), their new course will differ from the courses during preceding flight steps. The result will be that the birds follow curved routes as they migrate across longitudes. These routes are similar to great circle routes at high latitudes, but only if the birds use this compass at sunset (or sunrise), and not at other times of the day (see below and [48]).

Routes were simulated by assuming that the birds determine the departure direction at the time of sunset by adopting the orientation angle in relation to the observed sunset azimuth according to the time-compensated sunset compass at their initial departure site. Hence, orientation at the next flight step will change because of the combined effects of the change in sunset azimuth at the new site compared to the initial site and the change in the bird’s orientation in relation to the sun position because of the time difference from the initial departure site. Equivalent results would have been obtained under the alternative assumption that the birds established orientation at the new site at a fixed time according to the daily clock in phase with local time at the initial site, applying the orientation angle at this time in relation to the observed sun azimuth at the new site. Hence, the course for the next flight step would change because of the change in sun azimuth at the bird’s fixed departure time. It should be noted that it is not necessary to assume that the birds maintain their daily clock in phase with local time at the initial departure site throughout the migration, i.e., never resetting their daily clock during migration. The important condition is that the birds do not reset their circadian clock to local time between successive flight steps. They may adjust their daily clock and sun compass mechanism to new local time and solar conditions at a few stopover sites along the migration route and still continue along the same curved route, if they depart from a reset stopover site on the same true course as they had when arriving at this site [48] (see below).

#### Results: Characteristics of routes

**Fig. 1:**
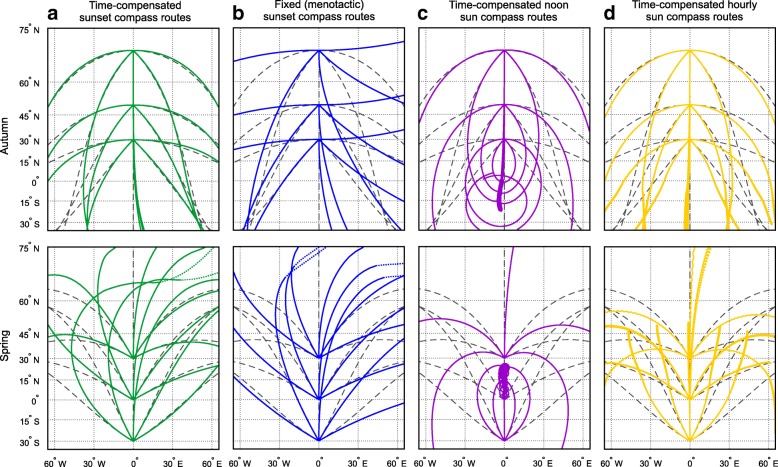
Simulated migration routes based on different sun compass mechanisms. **a** Time-compensated sunset compass orientation (green), **b** fixed (menotactic) sunset compass orientation (blue), and **c** time-compensated noon (pink) and **d** hourly sun compass orientation (yellow). The routes were calculated in daily steps of 200 km, with a new course for each step based on astronomical conditions at each daily departure location/time and assuming a constant geographic course within a step. In the case of the time-compensated hourly sun compass, a new direction was determined once an hour between 08:00 and 17:00, with steps of 20 km per hour. Autumn migration routes were simulated with 1 Sept as initial departure date and with initial departure directions of 90°, 135°, 180°, 225° and 270° from departure locations at latitudes 70°N, 50°N and 30°N. Spring migration were simulated with 1 April as departure date and with initial departure directions of 300°, 330°, 360°, 30° and 60° from departure locations at latitudes 30°S, Equator (0°) and 30°N. Dotted sections of sunset routes indicate situations where the sun did not set once the birds reached higher latitudes, thus where the lowest sun elevation was taken as reference instead. Great circle routes (dark grey dashed) are given for comparison to indicate the shortest routes. Since sun compass routes are independent of longitude, we show no maps here. The routes are plotted in a Mercator projection in which constant geographic courses (rhumblines or geographic loxodromes) are indicated as straight lines. See text for more details on simulations

Autumn routes are closely similar to great circle routes (Fig. 1a), hence the time-compensated sunset compass furnishes the birds with the means of great circle orientation. Agreement with great circle routes is large at high latitudes until equatorial latitudes are reached, where the agreement with great circle routes deteriorates, but total routes from higher to lower latitudes are still distance saving to a high degree. Spring migration routes starting at lower latitudes with an east component show a good agreement with great circle routes, while routes starting with a west component show significant deviations from the shortest route. Spring routes starting at lower latitudes on either side of (or at) the equator will be very sensitive to small differences in departure courses due to small differences in sunset directions over latitude and time in the tropics (see Additional file 3: Figure S3 for illustration).

Orientation with a time-compensated sunset compass is for obvious reasons problematic under polar conditions when the sun never sets below the horizon (dotted lines in Fig. 1a). A possible solution could be for the birds to use the lowest sun elevation instead of a true sunset. However, the lowest sun elevation is much more difficult to identify than sunset, so this might pose substantial problems. Additional problems arise when birds cross polar regions with a time-compensated sunset compass. The rapid changes in absolute directions that birds experience when flying across polar longitudes may result in sigmoid deflections of the routes near the North Pole (see Additional file 4: Figure S4A for illustration). Further problems with time-compensated sunset compass orientation are discussed in the last section.

### Fixed (menotactic) sunset compass

#### Assumptions

Birds following fixed (menotactic) sunset compass routes are assumed to orient at a fixed angle in relation to the local sunset azimuth throughout the migratory journey [43, 47, 49, 50]. Hence, the flight course will change according to the change in sunset azimuth along the bird’s migration route at the seasonal time of the bird’s passage. Also here, we focus on sunset, rather than sunrise, since the majority of passerines migrates at night. As in the case of the time-compensated sunset compass, this compass mechanism will be difficult to use during polar summers. Under polar summer conditions, we used the lowest sun elevation as reference instead, as we did in the case of the time-compensated sunset compass.

#### Results: Characteristics of routes

The routes resulting from fixed sunset compass orientation are in general closer to rhumbline than great circle routes (Fig. 1b). In the Northern Hemisphere the routes show a course change to the left (anticlockwise) in autumn (migration away from the pole) and to the right (clockwise) in spring (migration towards the pole). This holds also for movements from northerly latitudes continuing into the Southern Hemisphere, and for spring movements departing from the Southern Hemisphere towards northerly breeding latitudes. This means that birds migrating along the NE/SW axis will fly along courses that are shifting in a distance-saving way, whereas movements along the NW/SE axis will be in a distance-wasting way (see also below). However, the reverse pattern applies for autumn and spring migration in the Southern Hemisphere where routes along the NW/SE axis are closer to great circle routes (Southern Hemisphere seasons; not shown). Thus, the seasonal favourability of fixed sunset compass routes for migratory birds breeding in the Southern Hemisphere is the reverse of that for migratory birds breeding in the Northern Hemisphere. Birds reaching higher latitudes during spring migration relatively late in the season also face the problem that they will encounter midnight sun, thus where they have to resort to alternative means of identifying “sunset”, e.g., by using the lowest sun elevation instead.

### Time-compensated noon sun compass

#### Assumptions

Diurnal migrants could in theory use a time-compensated sun compass to establish their orientation around noon, a case that may not be very likely in passerine migrants, but that may be relevant for raptors that use thermal soaring flight. Such a time-compensated noon sun compass would allow the birds to compensate for the change in sun azimuth during the hours before and after noon according to the local conditions at the departure site. Routes were simulated assuming that the birds establish orientation at solar noon by changing their orientation angle in relation to the observed sun azimuth according to the time-compensated sun compass at the birds’ initial departure site. The principles are thus the same as for orientation with a time-compensated sunset compass, but routes will be quite different because of the difference in the apparent angular movement of the sun around noon compared to at sunset/sunrise (see [48]). Angular rates of change in sun azimuth are maximal at noon with large differences between latitudes, and azimuth changes are most accentuated at lower latitudes [48].

#### Results: Characteristics of routes

Routes curve in a distance-saving way at intermediate and higher latitudes in both autumn and spring, like the routes resulting from the time-compensated sunset compass (see above) (Fig. 1c). However, the routes associated with the time-compensated noon sun compass are close to great circle routes only at the highest latitudes, while they curve more strongly than great circle routes at moderately high and intermediate latitudes, thus being clearly longer than routes based on the time-compensated sunset compass. This difference is due to the differential rates of change of sun azimuth at noon versus sunset/sunrise as explained by Alerstam and Pettersson [48]. Furthermore, the time-compensated noon sun compass will break down at equatorial latitudes where the sun culmination occurs close to the zenith and where noon azimuth changes from southerly to northerly directions (or vice versa) with small changes in latitude/seasonal time. By way of example, serious complications occur for spring migration routes starting near the equator close to spring equinox, when the sun over the course of about 15 min changes from an easterly position during the morning to a westerly position in the afternoon (see Additional file 4: Figure S4B for illustration). This means that this compass will not be useful for migration in the latitude range between the Tropics of Cancer and Capricorn, neither in autumn nor in spring.

### Time-compensated hourly sun compass

#### Assumptions

Diurnal migrants may also use a time-compensated hourly sun compass, which differs from the former compasses in the assumption that orientation is not established only once each day (at noon), but at hourly intervals from 08:00 to 16:00 each day, with a 25 km advancement between hours. The birds are then assumed to change their orientation in relation to the observed sun azimuths at these hourly intervals according to the time-compensated sun compass at their initial departure site. It has been demonstrated that the sun compass mechanism among homing pigeons is flexible and takes into account differential changes of sun azimuth during different hours of the day [11, 38, 51], making this assumption about hourly orientation intervals not unreasonable.

#### Results: Characteristics of routes

Time-compensated hourly sun compass routes are similar to those based on a noon sun compass, except for the occurrence of a distinct daily curvature of tracks (Fig. 1d). The course changes anticlockwise during the daily migration period when the birds proceed southwards in autumn. This daily effect becomes gradually more pronounced as the birds reach successively lower latitudes. During spring, the daily course shifts are in the clockwise direction, increasing with increasingly northerly latitudes. The daily course changes become much exaggerated for migrants that have departed from equatorial latitudes, applying the time-compensated sun compass for these latitudes to the solar conditions at the higher latitudes. Hence, as for the noon sun compass, the hourly sun compass is useful only for migration at intermediate and higher latitudes in autumn as well as spring. It is interesting to note that regular course changes during the daily migration period (anticlockwise for movements away from the pole and clockwise for movement towards the pole in the northern Hemisphere, and vice versa in the Southern Hemisphere) are diagnostic for routes determined by this type of compass mechanism (may be revealed by analyses of high-resolution daily tracking data).

### Conclusions about feasibility of sun compass routes

We conclude and confirm that the time-compensated sunset compass, when used without correcting for the shift in local time as the bird moves along its migratory path, provides birds with a means of following distance-saving routes with shifting courses that are similar to great circle routes [48]. Such a compass strategy will work well during autumn migration and also during spring migration at mid- or high latitudes (> 30°N). However, we also demonstrate that using this compass strategy for northward spring migration out of the tropics has critical limitations, i.e., sensitivity to small differences in departure courses, often leading to distance-wasting routes (see Additional file 3: Figure S3). We therefore conclude that it is probably impractical for the birds to use the time-compensated sunset compass when departing from the tropics on spring migration. Only at higher latitudes will it be useful for them to adopt this compass mechanism during spring migration and follow close to great circle routes from there onwards to their final breeding destinations at high latitudes.

The feasibility of fixed (menotactic) sunset compass orientation depends on the migratory axis. During both spring and autumn migration, routes along the NE/SW axis in the northern Hemisphere and along the NW/SE axis in the Southern hemisphere are shifting in a distance-saving way. The use of time-compensated sun compass orientation based on the sun azimuth at noon or at hourly intervals during the day has significant limitations. Orientation with a time-compensated noon or hourly sun compass is therefore not feasible at all at lower latitudes on either side of the equator, neither during autumn nor spring migration.

## Simulations of bird migration routes based on different magnetic compass mechanisms

Birds can sense the Earth’s magnetic field and use the information for orientation [13, 14, 52, 53]. The avian magnetic compass is sensitive to the axial alignment, but not the polarity, of the magnetic field lines, thus birds determine the direction towards the magnetic equator or the closest magnetic pole using the inclination of the magnetic field lines [13]. They use the sign of the angle of inclination, i.e., whether the inclination is positive or negative, and not the exact angle of inclination, to distinguish equatorwards from polewards [14, 52, 54]. An alternative approach for birds to use magnetic field information for compass orientation is to fly along magnetoclinic compass routes [55]. In a pioneering and highly stimulating study, Kiepenheuer [55] suggested a magnetic compass mechanism based on the apparent angle of magnetic inclination, which is the projected angle of the inclination of the Earth’s magnetic field on a plane perpendicular to the movement direction of the bird (see Additional file 5: Figure S5 for an illustration and [55] for details on how to calculate the apparent angle of inclination). These magnetoclinic compass routes will change with changing inclination angles of the magnetic field along a birds’ migratory route and will lead the birds on shifting magnetoclinic compass courses in agreement with several cases of observed routes and experimental courses [30, 31, 55]. However, the hypothesis of magnetoclinic compass orientation has failed to gain any support from studies of magnetoreception mechanisms [14], nor from analyses of migration routes in the Arctic [26] or at a magnetic anomaly [56].

For the simulations of magnetic compass routes, we used the same initial departure directions (relative to geographic North) and a migration speed of 200 km/d as for the sun compass routes. Since magnetic field parameters are sensitive to both date and location, we set initial departure dates to 1 Sept 2010 for autumn and 1 April 2011 for spring migration, and calculated two versions of magnetic compass routes, one centered on the Palaearctic-African and the other on the Nearctic-Neotropic migration system. Magnetic field parameters were calculated with the Matlab script magdf.m by Maurice A. Tivey at Woods Hole Oceanographic Institution, USA, with the International Geomagnetic Reference Field (IGRF) model 2010 [57]. The apparent angles of inclination used for the magnetoclinic compass routes were calculated based on the angle of inclination of the Earth’s magnetic field at the initial departure location and the departure direction relative to magnetic North [55], and kept constant for the remainder of the route. When a bird reached a location where the angle of inclination was larger than the apparent angle of inclination, we assumed that it would follow the inclination isoclines by flying due magnetic east or west, as suggested by Kiepenheuer [55]. When the bird again reached a new location with an inclination angle smaller than the apparent angle of inclination, it continued along the magnetoclinic compass route. Since we assumed that the birds read their compass only once a day, every 200 km, and not constantly, as assumed by Kiepenheuer [55], no resetting of the compass was necessary in our simulations for the birds to get back to areas with inclination angles smaller than the apparent angle of inclination.

### Fixed (menotactic) magnetic compass

#### Assumptions

Migratory birds using a magnetic compass for orientation follow a constant magnetic compass course which will lead them along trajectories with a changing geographic course. The discrepancy between constant magnetic and constant geographic compass courses occurs because the poles of the Earth’s magnetic field do not coincide with the geographic poles, and the horizontal polarity of the magnetic field varies over space and time, which results in a changing relationship between the geographic and magnetic reference systems [15, 57]. Trajectories following fixed magnetic compass routes (magnetic loxodromes) will therefore vary with magnetic declination, i.e., the difference between the magnetic and geographic direction at a specific location and time, and will depend strongly on the geographic region a bird is crossing [18, 26].

#### Results: Characteristics of routes

**Fig. 2:**
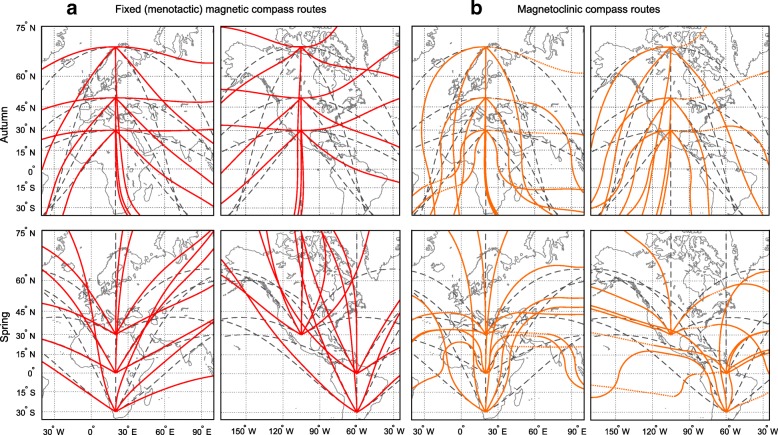
Simulated migration routes based on different magnetic compass mechanisms. **a** Fixed (menotactic) magnetic compass (red) and **b** magnetoclinic compass orientation (orange). The routes are calculated in daily steps of 200 km, determining a new course for each step based on geomagnetic conditions at each daily departure location and assuming a constant geographic course within a step. Autumn migration routes were simulated with 1 Sept 2010 as initial departure date, spring migration routes with 1 April 2011 as departure date. For each compass mechanism, routes were centred on the Palaearctic-African (left panels) and Nearctic-Neotropic (right panels) migration systems, respectively. Dotted sections of magnetoclinic compass routes indicate situations where the angle of inclination of the Earth’s magnetic field was larger than the apparent angle of inclination, thus where a magnetoclinic compass could not be used, and the birds instead were assumed to orient a fixed magnetic compass instead. Great circle routes (dark grey dashed) are given for comparison to indicate the shortest routes. All maps are in Mercator projection. See Fig. 1 and text for more details

In general, fixed magnetic compass routes within the Palaearctic-African migration system run closer to great circle routes than the routes within the Nearctic-Neotropic migration system (Fig. 2a). Within the Palaearctic-African migration system, fixed magnetic compass routes starting in the Northern Hemisphere during autumn migration follow great circle routes more closely than spring routes starting in the southern hemisphere. The reverse is true for birds migrating within the Nearctic-Neotropic migration system. Here, spring routes along fixed magnetic compass courses are generally closer to great circle routes than autumn routes, specifically if the birds depart from wintering areas in South America.

It has to be emphasized that trajectories based on fixed magnetic compass courses depend on the properties of the Earth’s magnetic field at the departure location and along the migratory routes, thus they can vary considerably between sites and over time, especially close to the magnetic poles where differences in magnetic declination can be large between nearby locations [18, 26, 58].

### Magnetoclinic compass

#### Assumptions

Birds are assumed to follow the fixed apparent angle of inclination determined at the initial departure location, as long as the inclination angle of the Earth’s magnetic field remains smaller than the apparent angle of inclination [55]. Birds following such a magnetoclinic compass route will change direction when the inclination angle and magnetic declination change along the route [48]. Magnetoclinic compass routes therefore depend on the initial departure settings (magnetic inclination at departure location and initial departure direction relative to magnetic North), as well as the properties of the Earth’s magnetic field (magnetic inclination and declination) along the route. One common feature for all magnetoclinic compass routes is that they cross the magnetic equator (where magnetic inclination is 0°) orienting due north or south relative to magnetic North. An important restriction for the use of a magnetoclinic compass is that it cannot be used when the absolute value of the angle of inclination at a location along the route becomes larger than the fixed apparent angle of inclination [55]. In such situations, we assumed that the birds would follow the inclination isoclines by flying due magnetic east or west, as suggested by Kiepenheuer [55].

#### Results: Characteristics of routes

Magnetoclinic compass routes during autumn follow great circle routes more closely within the Nearctic-Neotropic migration system compared to the Palaearctic-African migration system (Fig. 2b). However, these differences are rather small and depend highly on location within the migration system. During spring migration, the majority of magnetoclinic compass routes in both migration systems vary considerably in not very favourable ways. The outcome of the routes during spring is also highly sensitive to small differences in the initial departure directions, and thereby the apparent angle of inclination (see Additional file 6: Figure S6 for illustration), requiring a highly sensitive compass. In many cases, the birds reached areas with angles of magnetic inclination larger than the apparent angle of inclination (dotted lines in Fig. 2b), forcing them to follow magnetic inclination isoclines during parts or the entire migration.

### Conclusions about feasibility of magnetic compass routes

We conclude that fixed magnetic compass routes are generally more feasible than magnetoclinic compass routes. Fixed magnetic compass routes run closer to great circle routes, and are thereby more distance saving, within the Palaearctic-African than within the Nearctic-Neotropic migration system. As the use of fixed magnetic compass routes depends on the local magnetic declination along the migration route, it varies over both space and time, thus the trajectories differ between geographic areas.

The trajectories of the magnetoclinic compass routes depend on both the inclination and declination of the Earth’s magnetic field, which makes them more unpredictable and susceptible to variations of the magnetic field than fixed magnetic compass routes. It cannot be excluded, however, that magnetoclinic routes might be feasible in the case of specific bird populations.

## Comparison of simulated routes for three populations of passerine migrants

In this second series of analyses, we compared autumn and spring compass routes for three examples of nocturnal long-distance migrants, northern wheatears, migrating from Greenland to western Africa, and pied flycatchers and willow warblers migrating from northern Scandinavia to western and eastern Africa, respectively. We calculated autumn and spring migration routes based on different compass mechanisms between specified departure and destination locations. As in the previous sections, we calculated the routes in daily steps of 200 km, determining a new course for each step based on astronomical and geomagnetic conditions at each daily departure location/time, and assuming a constant geographic course within a step. We intentionally chose the initial departure directions so that the birds successfully reached their destination. In the case of the northern wheatears migrating across the North Atlantic, stopovers are of course not possible over the open ocean, but for simplicity and to be able to compare the different routes between populations, we assumed the same rules for all of them. Autumn migration routes were simulated with 1 August 2010 and spring migration with 1 April 2011 as initial departure date, respectively.

The following five compass routes were calculated between the specified breeding and wintering locations: (1) time-compensated sunset compass route (see assumptions and characteristics above); (2) fixed (menotactic) sunset compass route (see assumptions and characteristics above); (3) fixed (menotactic) magnetic compass route (see assumptions and characteristics above); (4) magnetoclinic compass route (see assumptions and characteristics above); (5) rhumbline (loxodrome) route, which birds may follow if they use a star compass sensu [9] (no time compensation), a time-compensated sunrise/sun/sunset compass where the birds reset their circadian clock in phase with the new local conditions at each step of orientation, or a magnetic compass regularly calibrated by averaging polarized skylight information at sunrise and sunset, as proposed by [19, 22, 23, 59]. We used the exact great circle route (orthodrome), i.e., the shortest route between two locations on Earth, as reference of comparison for the other routes. Since we compared nocturnal passerine migrants which usually depart at or shortly after sunset (cf. [60, 61]), we did not consider sun compass mechanisms based on sun observations at noon or during the day (time-compensated noon and time-compensated hourly sun compass).

### Results: Characteristics of routes

**Fig. 3:**
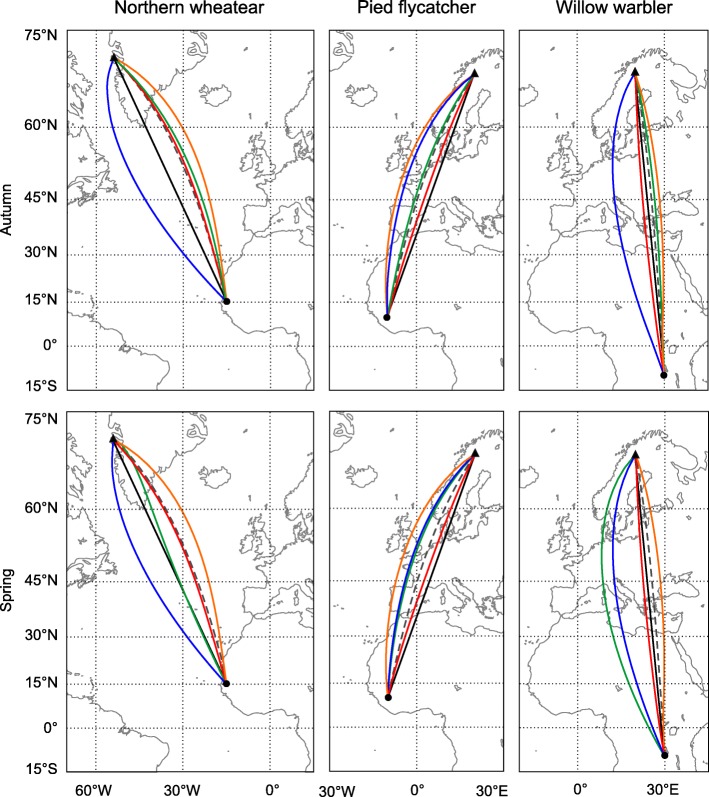
Simulated autumn and spring migration routes of three populations of songbirds. Migration routes for populations of northern wheatears migrating from Greenland to their wintering areas in western Africa, and pied flycatchers and willow warblers, migrating from northern Scandinavia to their wintering areas in western and eastern Africa, respectively. Autumn migration routes were simulated with 1 Aug 2010 as initial departure date, spring migration routes with 1 April 2011 as departure date. Illustrated are the rhumbline routes (black), time-compensated sunset compass routes (green), fixed (menotactic) sunset compass routes (blue), fixed (menotactic) magnetic compass routes (red), and magnetoclinic compass routes (orange). The exact great circle routes (dark grey dashed) are shown for comparison. Initial departure locations are indicated as black triangles and destinations as black dots. All maps are in Mercator projection. See Table 1 for details

The migratory axes of the three populations in Fig. 3 are all rather close to N/S, making the differences in distance between the rhumbline and great circle routes small (≤1%; Table 1). Several of the compass mechanisms provide efficient trajectories with distance ratios exceeding unity with only a minor amount (≤1%). This holds true for all time-compensated sunset compass routes, with the exception of willow warblers which fly an extra distance of around 6% compared to the great circle route during spring migration. Distance ratios of fixed sunset compass routes, on the other hand, are only within 1% of unity in the case of the pied flycatcher (case with NE/SW migratory axis). For systems with a NW/SE migration axis, like the northern wheatear and willow warbler, trajectories based on fixed sunset orientation may incur up to 9% extra distance, both in autumn and in spring (Fig. 3; Table 1). Such levels of extra costs may be important, thus there may be significant selection against the use of the time-compensated sunset compass during spring migration as well as against the use of a fixed sunset compass in both seasons for populations with a NW/SE axis.

**Table 1:** Distance ratios in relation to the shortest great circle distance for simulated trajectories based on different compass mechanisms in three examples of songbird migration systems (shown in Fig. 3)

	Breeding location	Wintering location	Distance of great circle route (km)	Autumn + Spring			Autumn		Spring	
				Rhumbline route	Fixed (menotactic) magnetic compass route	Magnetoclinic compass route	Time-comp. sunset compass route	Fixed (menotactic) sunset compass route	Time-comp. sunset compass route	Fixed (menotactic) sunset compass route
Northern wheatear	70° N, 54° W	15° N, 15° W	6672	1.01	1.00	1.01	1.00	1.09	1.01	1.07
Willow warbler	68° N, 20° E	10° S, 30° E	8710	1.00	1.00	1.00	1.00	1.04	1.06	1.04
Pied flycatcher	68° N, 20° E	10° N, 10° W	6814	1.01	1.00	1.02	1.00	1.00	1.00	1.01

The rhumbline route, the route associated with a fixed (menotactic) magnetic compass course and the magnetoclinic compass route do not differ between seasons, thus the distance ratios are given only once. For the other two compass mechanisms (time-compensated and fixed (menotactic) sunset compass) trajectories differ between autumn and spring seasons with different distance ratios as given in the table. Distance ratios (always > 1) are rounded to two decimals, meaning that ratios between 1.000 and 1.0049 are given as 1.00

For the case of fixed magnetic compass routes, the routes of the three populations all fall within a geographic zone where the trajectories curve in a distance-saving way in relation to the rhumbline (a zone extending from Greenland and across Europe; see [62]). Magnetoclinic compass routes for northern wheatears and willow warblers lie within < 1% distance of the great circle route, while pied flycatchers take an about 2% longer route. Since the efficiencies of these routes highly depend on the global pattern of magnetic declination and inclination, they do not apply everywhere. In other zones, e.g., in North America, routes along magnetic loxodromes and magnetoclinic routes will curve in an unfavourable way, leading to extra distances (see Fig. 2 above). This means that the use of fixed magnetic compass and magnetoclinic compass orientation may be selected against in some zones, but selectively promoted in other zones [18, 62].

### Conclusions about feasibility of different compass routes

Overall, all compass mechanisms examined in the three selected examples in Fig. 3 successfully led the birds from the breeding to the non-breeding areas, and back again, without the need for a resetting of the compass settings or change to alternative compass mechanisms. Thus, we would like to stress that all compass mechanisms are feasible for the populations in Fig. 3, and that the costs for using one rather than the other mechanism are rather small. This illustrates that for many migratory routes all or several compass mechanisms would successfully guide birds to their migratory destination. Still, it is important to note that such evaluations of migration routes highly depend on the geographic location, migratory season, and migratory axis (NE/SW or NW/SE), thus the findings cannot be generalized beyond these examples. In the next section, we therefore modelled compass routes for a population of extreme long-distance migrants, northern wheatears breeding in Alaska and migrating to eastern Africa, where the different compass courses differ much more, making it more likely to find biological significant differences between the courses and the route selected by the birds.

## Possible compass mechanisms used by northern wheatears breeding in Alaska

In this third series of analyses, we investigate which compass routes most closely match the actual routes taken by northern wheatears migrating between the breeding sites in Alaska and wintering sites in eastern Africa during autumn and spring. We compare the five compass strategies described in the previous sections with the actual routes of five individual birds estimated from light-level geolocation information [32]. For the route simulations, we used the individual birds’ actual departure and arrival locations and departure and arrival dates from the breeding and wintering sites, respectively. For each individual, we calculated the daily migration flights by dividing departure and arrival dates by the number of days the bird spent on migration (see [32] for details). As in the earlier described simulations, we determined a new course for each step based on astronomical and/or geomagnetic conditions at each daily departure location/date, and assumed a constant geographic course within a step. For simplicity, we estimated the distance of the actual migration route of the northern wheatears as the cumulative distance following fixed sunset compass routes between the migratory starting point, every stopover and the migratory destination (“actual migration route” hereafter). Since the accuracy and precision of the location estimates obtained from the geolocators are not exact, it should be kept in mind that the distances for these actual migration routes are rough approximations. Also, since the location estimates from the light-level geolocators are missing at high latitudes in spring because of the dependency of the tracking method on sunrise and sunset times, migration distance was calculated from the actually tracked route plus the distance of the birds’ last location estimate to the breeding area in Alaska (Table 2). Deviations from this simplified course are not incorporated here, so that the actual migration routes are somewhat shorter than the “true” migration distances capturing the birds’ migratory movement in more detail (cf. [32]).

**Table 2:** Distance ratios in relation to the distance of the actual migration route, as calculated in this study, for simulated trajectories based on different compass mechanisms in five individuals of northern wheatear migrating between Alaska and Eastern Africa, as revealed by light-level geolocation (cf. [32]); see Fig. 4)

Autumn migration	Departure location	Arrival location	Distance of actual route (km)	Great circle route	Rhumbline route	Time-comp. sunset compass route	Fixed (menotactic) sunset compass route	Fixed (menotactic) magnetic compass route	Magnetoclinic compass route
B070	66°N, 145°E	13°N, 37°E	12840	0.88	1.17	0.88	1.01	1.15	–
E552	65.5°N,145.4°E	7°N, 30°E	14440	0.83	1.08	0.83	0.96	1.11	–
E553	68.6°N, 149.5°E	3°N, 30°E	13970	0.87	1.14	0.88	0.99	1.13	–
B801	65°N, 145°E	8°N, 34°E	13950	0.85	1.13	0.85	0.99	1.12	–
B823	65°N, 146°E	12°N, 31°E	14160	0.81	1.08	0.81	0.94	1.09	–
Spring migration	Departure location	Arrival location	Distance of actual route (km)	Great circle route	Rhumbline route	Time-comp. sunset compass route	Fixed (menotactic) sunset compass route	Fixed (menotactic) magnetic compass route	Magnetoclinic compass route
B070	12°N, 40°E	65.5°N,145.4°E	13310	0.86	1.13	0.92	0.98	1.11	0.99
E552	5°N, 30°E	66°N, 145°E	14270	0.85	1.10	0.94	0.97	1.14	1.01
E553	3°N, 30°E	68.6°N, 149.5°E	13450	0.90	1.19	0.98	0.98	1.19	1.04
B801	9°N, 36°E	65°N, 145°E	13370	0.88	1.18	0.95	1.00	1.15	1.03
B823	6°N, 31°E	65°N, 146°E	13620	0.89	1.17	0.96	1.03	1.19	1.04

### Results: Characteristics of routes

**Fig. 4:**
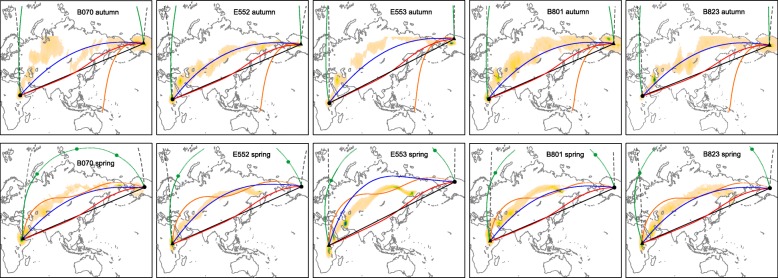
Simulated autumn and spring migration routes of northern wheatears migrating breeding in Alaska. Migration routes are shown between the breeding sites in Alaska and the wintering areas in eastern Africa in comparison to the actual routes taken by the individual birds as estimated from geolocator information [see 32 for details on tracks]. Illustrated are the rhumbline routes (black), time-compensated sunset compass routes (green), fixed (menotactic) sunset compass routes (blue), fixed (menotactic) magnetic compass routes (red), and magnetoclinic compass routes (orange). The exact great circle routes (dark grey dashed) are shown for comparison. Initial departure locations are indicated as black triangles and destinations as black dots. Green dots indicate locations where the compass courses were reset. Dotted sections of time-compensated sunset compass routes indicate locations where the sun did not set below the horizon, thus where the birds had to use the lowest sun elevation as sunset. Dotted sections of magnetoclinic compass routes indicate locations where the angle of inclination of the Earth’s magnetic field was larger than the apparent angle of inclination. Estimates of locations, incl. 95% credible intervals are given in yellow to green shades (see [32] for details). All maps are in Mercator projection. For further information see Table 2 and main text

The fixed (menotactic) sunset compass routes provide the best fit to the actually flown routes by the individual wheatears as estimated from light-level geolocation information during both autumn and spring migration, (Fig. 4). These routes also most closely match the distances flown by the birds, with average distance ratios closest to the distance of the actual routes (Table 2).

The trajectories of the time-compensated sunset compass routes run close to the great circle across the Arctic Ocean past the North Pole during autumn, thus much farther north than the actual migration routes. It was not possible to simulate time-compensated sunset compass routes during spring migration without resetting the compass at least once, since the movement of the equatorial sun near spring equinox used as reference led to sudden, large shifts in direction towards the end of the journey (see Additional file 7: Figure S7 for illustration). However, as mentioned earlier, the birds can circumvent this problem by resetting their inner clock to local time and use the local sun ephemeris as reference for the sun compass at a few selected stopover sites along the migration route [48]. When departing from one of these sites on the same compass course as arriving to that site, the birds will continue along the same curved route as before the stopover, with a minor effect associated with the lack of course change at this site. In our simulations we therefore introduced such a resetting of the inner clock and compass setting whenever the difference between sunset at the initial departure location and the current location exceeded 45°. During autumn and spring migration, birds flying along time-compensated sunset compass routes also encounter the midnight sun at high latitudes (in autumn at latitudes > 80° and therefore not visible in Fig. 4; dotted lines in spring. For these and other reasons (barrier crossing over long distances of the Arctic Ocean), time-compensated sunset compass routes are therefore not very feasible, even though they would guide the birds along the shortest routes between the breeding and wintering sites.

Both the rhumbline and fixed magnetic compass routes run along far more southerly courses than estimated by the light-level geolocation data. Following along rhumbline routes would involve the crossing of the Bering Sea, the Sea of Okhotsk, and the Arabian Sea, while fixed magnetic compass routes results in the crossing of the Sea of Okhotsk and the Arabian Sea. While such sea crossings per se are not expected to pose any major problems for migrants like northern wheatears, the estimated tracks do not indicate that the northern wheatears flew along any of these trajectories.

Autumn trajectories of the magnetoclinic compass routes consistently led the birds in all five examples immediately southwards (Fig. 4). This phenomenon is caused by the distribution of the magnetic inclination isoclines and magnetic declination in this area, making the use of magnetoclinic routes from these locations highly unlikely. However, departure from more westerly locations leads the birds along the magnetic inclination isoclines (see Additional file 8: Figure S8 for illustration), bringing them westwards, as suggested by earlier studies [29, 31, 55]. During spring migration, the trajectories along the magnetoclinic compass route successfully led all birds to their destination. In all cases, however, the local inclination angle exceeded the apparent angle of inclination when the birds reached eastern Russia, forcing them to fly the final section of their journey along the inclination isocline.

### Conclusions about feasibility of possible compass routes used by northern wheatears breeding in Alaska

Clearly, in the case of the northern wheatears breeding in Alaska, fixed (menotactic) sunset compass routes most closely agree with the actual routes flown by the individual birds as estimated from light-level geolocation information. During both autumn and spring migration, birds orienting at a fixed angle relative to local sunset, determined at the departure location, will successfully reach their migratory destination without the need to reset the compass at any time during the journey. They will follow trajectories that are very close to the actually flown routes in location and distance (Fig. 4; Table 2). Still, as outlined earlier, the feasibility of fixed sunset compass orientation depends on the migratory axis. In the northern Hemisphere, birds like the northern wheatears from Alaska migrating along the NE/SW axis will fly along courses that are shifting in a distance-saving way. This is not the case for birds migrating along a NW/SE axis, which should be kept in mind when making general conclusions about the use of compass mechanisms.

Previous studies modelling constant compass courses for the migration routes of northern wheatears breeding in Alaska did not include fixed sunset compass routes in their models, and both found that the magnetoclinic compass routes spatially coincided best with the actual routes of the birds [29, 31]. Both studies, however, used different departure locations and dates than we used in the current study, and the simulations by Åkesson and Bianco [31] included a resetting of the apparent angle of inclination along the autumn migration route, which explains some of the discrepancies between the different studies. Irrespective of these differences, there is currently no experimental evidence that birds are able to sense the apparent angle of inclination, thus this model still lacks an empirical background [29, 55, 58]. Together with the problem of using a magnetoclinic compass in areas with local angles of magnetic inclination exceeding the apparent angle of inclination, it is therefore less likely that the northern wheatear breeding in Alaska use a magnetoclinic compass for orientation, but that these populations use a fixed sunset compass courses instead.

## Conclusions

The feasibility of different compass mechanisms varies greatly with latitude, migratory direction, and migration season. In the case of the magnetic compass mechanisms, the magnetic field properties at different geographic location are the main factors that determine the course of the routes. Our simulations in the first section show that there is little support for the use of a time-compensated noon or hourly sun compass by diurnal migrants, especially not at lower latitudes and for longer journeys. Time-compensated and fixed sunset compass routes on the other hand may be feasible, but primarily at higher latitudes (time-compensated sunset compass) or along the NE/SW axis (fixed sunset compass in Northern Hemisphere). The feasibility of the two magnetic compasses depends on geographic location, with the magnetoclinic compass further being restricted to areas with lower angles of inclination.

Nevertheless, as shown in Fig. 3, there are areas on Earth where all compass mechanisms may be used by different populations of migrants without inflicting too large deviations from the optimal routes. This, however, does not mean that they should randomly switch from one compass course to another depending on the availability of orientation cues, as this might lead to substantial detours (see supplement in [59]). Instead, the birds should follow one compass course and regularly calibrate different compass cues (solar, stellar, magnetic) with each other in order to be able to use both magnetic and celestial compass information during the actual flight [19, 22, 63].

In the case of the northern wheatears from Alaska our simulations show that there is only one compass course, following fixed sunset compass orientation, which fits well with the realized migration tracks of free-flying birds. All other compass routes involve substantial detours and lead the birds along trajectories far from the known tracks. This suggests that the birds might indeed follow fixed sunset compass orientation, and recalibrate their other compass cues relative to this information.

It is reasonable to assume that different bird populations use the compass mechanism that brings them to their destination with as few changes as possible in the compass settings. Also, it is probably less likely to assume that a bird migrating along one of the compass courses will switch to an entirely different mechanism in the middle of the migration, but rather reset the current course to a new start direction. As mentioned above, this does not mean that the birds do not use different compass cues to determine their departure direction, thus that they still recalibrate the different compass cue with each other to be able to switch between them, if necessary, for example when weather conditions change.

Taken together, routes following a single compass course throughout the migratory journey might not be very common, thus birds of many populations likely have to reorient once to a few times along the migration route to successfully reach their destination. Such pre-programmed directional changes at specific locations along the migration route have been experimentally demonstrated in several bird populations (cf. [3, 33, 34]). In addition, there is growing evidence that birds use map information to navigate to their migratory destination already during their first return migration during spring [6, 37]. It should also be kept in mind that several factors besides compass mechanisms may affect migratory routes at both proximate and ultimate levels. Distributions of resources and habitats, along with topographical features and wind conditions, will determine which routes are optimal. In addition, navigation capability and responses to wind drift are also important determinants of migration routes (e.g. [30, 62, 64–66]). This means that the course control of migratory birds may be so complex and variable (violating the assumption of constant orientation according to a single compass mechanism) that it will be difficult to identify probable compass mechanisms from the geometry of the observed routes. On the other hand, the possibilities for critical comparisons between predicted theoretical trajectories and observed routes have improved with the recent and ongoing tracking revolution in the animal migration field, where novel techniques provide much new and precise information about travel routes of individual animals (e.g. [32, 67–73]).

## Additional files

Additional file 1: **Figure S1.** Effect of daily travel distance on flight trajectories of migrants following time-compensated sunset and fixed (menotactic) sunset compass routes. The routes were calculated in daily steps of 100 km (blue), 200 km (green), and 300 km (red) with a new course for each step based on astronomical conditions at each daily departure location/time and assuming a constant geographic course within a step. Autumn migration routes were simulated with 1 Sept as initial departure date and with initial departure directions of 90°, 135°, 180°, 225° and 270° from departure locations at latitudes 70°N. Spring migration were simulated with 1 April as departure date and with initial departure directions of 300°, 330°, 360°, 30° and 60° from departure locations at latitudes 30°S. Dotted sections of routes indicate situations where the sun did not set anymore once the birds reached higher latitudes, thus where the lowest sun elevation was taken as reference instead. Great circle routes (dark grey dashed) are given for comparison to indicate the shortest routes. The routes are presented in Mercator projection. (PDF 371 kb)

Additional file 2: **Figure S2.** Effect of time of season on flight trajectories of migrants following time-compensated sunset and fixed (menotactic) sunset compass routes. Autumn migration routes were simulated with 1 Aug (blue), 1 Sept (green), and 1 Oct (red) as initial departure dates and with initial departure directions of 90°, 135°, 180°, 225° and 270° from departure locations at latitudes 70°N. Spring migration were simulated with 1 March (blue), 1 April (green), and 1 May (red) as departure dates and with initial departure directions of 300°, 330°, 360°, 30° and 60° from departure locations at latitudes 30°S. All routes were calculated in daily steps of 200 km with a new course for each step based on astronomical conditions at each daily departure location/time and assuming a constant geographic course within a step. Dotted sections of routes indicate situations where the sun did not set anymore once the birds reached higher latitudes, thus where the lowest sun elevation was taken as reference instead. Great circle routes (dark grey dashed) are given for comparison to indicate the shortest routes. The routes are presented in Mercator projection. (PDF 357 kb)

Additional file 3: **Figure S3.** Time-compensated sunset compass routes during spring migration with initial departure directions of 354°, 356°, 358°, 0°, 2°, 4° and 6°. Spring routes starting at lower latitudes on either side of (or at) the equator are very sensitive to small differences in departure courses due to small differences in sunset directions over latitude and time in the tropics. Great circle routes (dark grey dashed) are given for comparison to indicate the shortest routes. The routes are presented in Mercator projection. (PDF 120 kb)

Additional file 4: **Figure S4.** (A) The time-compensated sunset compass route is deflected near the geographic North Pole because of the rapid changes in absolute directions that the bird is experiencing when flying across longitudes near the poles. Red crosses give the positions of a putative bird departing from Alaska along a time-compensated sunset compass route towards the North Pole, reorienting every 200 km. Gnomonic map projection. (B) Sun position (azimuth) at the equator (0° latitude, 0° longitude) over a 24-h period on spring equinox (21 March). Birds starting near the equator close to spring equinox on a time-compensated sunset compass route will run into problems because of the sudden shift of the sun from the east to the west over the course of about 15 min. See also Additional file 6: Figure S6. (PDF 141 kb)

Additional file 5: **Figure S5.** Visualisation of the magnetoclinic compass. Magnetoclinic orientation refers to the case where migratory birds fly at a constant “apparent angle of inclination” (γ^′^ in blue). The apparent angle of inclination is the inclination of the geomagnetic field projected on a plane orthogonal to the bird’s heading or body axis. As inclination changes with latitude, a migrant must change its course in order to keep γ^′^ constant. In horizontal flight the apparent angle of inclination is a function of the geomagnetic inclination (γ in red) and the bird’s flight course (α in green), according to the relationship tan(γ^′^) = tan(γ)/ sin(α). The illustration shows the headings of a bird flying along a fixed γ’ in areas with different angles of inclination γ_1_ (left graph) and γ_2_ (right graph). The bird maintains a fixed γ^′^ by adjusting its heading from more westerly directions α_1_ to more southerly directions α_2_ with decreasing geomagnetic inclination from γ_1_ (left graph) and γ_2_ (right graph). Magnetoclinic orientation will be affected if birds do not fly horizontally and also by wind conditions depending on whether the birds perceive the apparent inclination magnetostatically in relation to their body axis or by a magnetic induction process in relation to their trajectory through the magnetic field, as evaluated by Alerstam (1987: J Exp Biol. 1987;130:63–86). These effects are not included in the simplified geometric explanation in the figure here. (PDF 173 kb)

Additional file 6: **Figure S6.** Two examples of magnetoclinic compass routes during spring migration starting from the equator (0° latitude; left graph) or 20°S (right graph) with initial departure directions of 354°, 356°, 358°, 0°, 2°, 4° and 6°. Great circle routes (dark grey dashed) are given for comparison to indicate the shortest routes. The routes are presented in Mercator projection. (PDF 171 kb)

Additional file 7: **Figure S7.** Explanation for why birds starting from equatorial latitudes during spring migration may not reach their destinations if following a time-compensated sunset compass. Example of a bird departing on spring migration in eastern Africa (12°N, 20°E; blue triangle) on 14 April 2014 towards its destination in Alaska (black dot), advancing 295 km/d. Graphs on the right show the sun ephemeris curves, i.e. the azimuth of the sun relative to Universal time, for three consecutive days illustrated in red, green and turquoise, incl. local sunset (dots in respective colours). The blue ephemeris curves give the azimuth of the sun at the departure location and departure date, which the bird uses as reference. The blue triangles show the sun azimuth at the departure location at the time of local sunset for each of the three days. The bird determines its departure direction at local sunset, but uses the sun ephemeris from the departure location and departure date as reference. Thus, it changes its daily departure direction by the difference between the local sunset azimuth and the azimuth of the sun at that specific time at the departure location and date. The sudden shift in compass direction is the result of the sun changing its position relatively quickly from west to south to east at noon at the departure location. These shifts are most dramatic at the geographic equator, thus affects birds departing from areas close to the equator, and migrate enough days for the local sunset time to coincide with the time of noon at the departure location. Birds can avoid this by updating their inner clock at least once along their journey at higher latitudes and then continue using the sun ephemeris of the reset location as reference for the remaining journey. The map is in Mercator projection. (PDF 236 kb)

Additional file 8: **Figure S8.** (A) Magnetoclinic compass routes of a northern wheatear (B070) departing from 66°N at different longitudes (155° E, 160° E, 175° W, 160° W, 155° W; black triangles) in westerly directions (270° relative to magnetic North). Because of the different angles of magnetic inclination at the different starting locations (γ = 79.1°, 76.9°, 75.5°, 75.2°, 76.2° from easterly to westerly sites), the bird starts with different apparent angles of inclination (γ^′^ = γ). Depending on the distribution of magnetic inclination, the birds are either led immediately southwards (solid lines, where γ^′^ > γ) or along the magnetic inclination isoclines (dashed lines, where γ > γ^′^). (B) Magnetoclinic compass routes of the same bird starting from its initial departure location with different γ^′^. It is possible for the bird to reach its destination (black dot at 13°N, 37°E) by using a magnetoclinic compass and without resetting the compass along the journey, but the path is highly sensitive to minute changes of the apparent angle of inclination (sensitivity < 2 × 10^− 8^ deg.), making this strategy highly unlikely. The maps are in Mercator projection. (PDF 388 kb)
