# Supplementary material for: Feasibility of sun and magnetic compass mechanisms in avian long-distance migration
Source: Mov Ecol. 2018 Jun 6;6:8. doi: 10.1186/s40462-018-0126-4 (PMC5989362; doi:10.1186/s40462-018-0126-4)
Supplement: Supplementary file 1 — Figure S1. Effect of daily travel distance on flight trajectories of migrants following time-compensated sunset and fixed (menotactic) sunset compass routes. The routes were calculated in daily steps of 100 km (blue), 200 km (green), and 300 km (red) with a new course for each step based on astronomical conditions at each daily departure location/time and assuming a constant geographic course within a step. Autumn migration routes were simulated with 1 Sept as initial departure date and with initial departure directions of 90°, 135°, 180°, 225° and 270° from departure locations at latitudes 70°N. Spring migration were simulated with 1 April as departure date and with initial departure directions of 300°, 330°, 360°, 30° and 60° from departure locations at latitudes 30°S. Dotted sections of routes indicate situations where the sun did not set anymore once the birds reached higher latitudes, thus where the lowest sun elevation was taken as reference instead. Great circle routes (dark grey dashed) are given for comparison to indicate the shortest routes. The routes are presented in Mercator projection. (PDF 371 kb) [file 40462_2018_126_MOESM1_ESM.pdf]

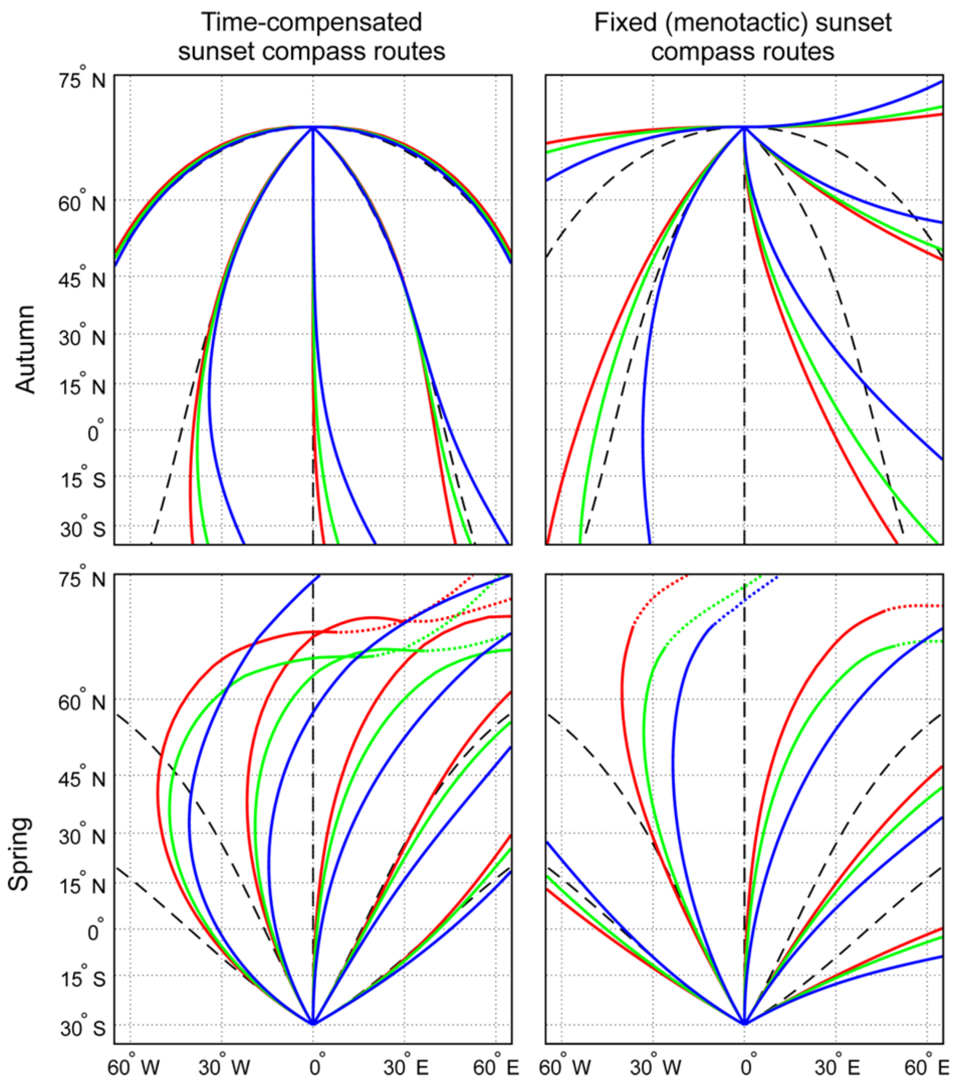

Figure S1. Effect of daily travel distance on flight trajectories of migrants following time-compensated sunset and fixed (menotactic) sunset compass routes. The routes were calculated in daily steps of 100 km (blue), 200 km (green), and 300 km (red) with a new course for each step based on astronomical conditions at each daily departure location/time and assuming a constant geographic course within a step. Autumn migration routes were simulated with 1 Sept as initial departure date and with initial departure directions of 90°, 135°, 180°, 225° and 270° from departure locations at latitudes 70°N. Spring migration were simulated with 1 April as departure date and with initial departure directions of 300°, 330°, 360°, 30° and 60° from departure locations at latitudes 30°S. Dotted sections of routes indicate situations where the sun did not set anymore once the birds reached higher latitudes, thus where the lowest sun elevation was taken as reference instead. Great circle routes (dark grey dashed) are given for comparison to indicate the shortest routes. The routes are presented in Mercator projection.
