# Supplementary material for: Feasibility of sun and magnetic compass mechanisms in avian long-distance migration
Source: Mov Ecol. 2018 Jun 6;6:8. doi: 10.1186/s40462-018-0126-4 (PMC5989362; doi:10.1186/s40462-018-0126-4)
Supplement: Supplementary file 2 — Figure S2. Effect of time of season on flight trajectories of migrants following time-compensated sunset and fixed (menotactic) sunset compass routes. Autumn migration routes were simulated with 1 Aug (blue), 1 Sept (green), and 1 Oct (red) as initial departure dates and with initial departure directions of 90°, 135°, 180°, 225° and 270° from departure locations at latitudes 70°N. Spring migration were simulated with 1 March (blue), 1 April (green), and 1 May (red) as departure dates and with initial departure directions of 300°, 330°, 360°, 30° and 60° from departure locations at latitudes 30°S. All routes were calculated in daily steps of 200 km with a new course for each step based on astronomical conditions at each daily departure location/time and assuming a constant geographic course within a step. Dotted sections of routes indicate situations where the sun did not set anymore once the birds reached higher latitudes, thus where the lowest sun elevation was taken as reference instead. Great circle routes (dark grey dashed) are given for comparison to indicate the shortest routes. The routes are presented in Mercator projection. (PDF 357 kb) [file 40462_2018_126_MOESM2_ESM.pdf]

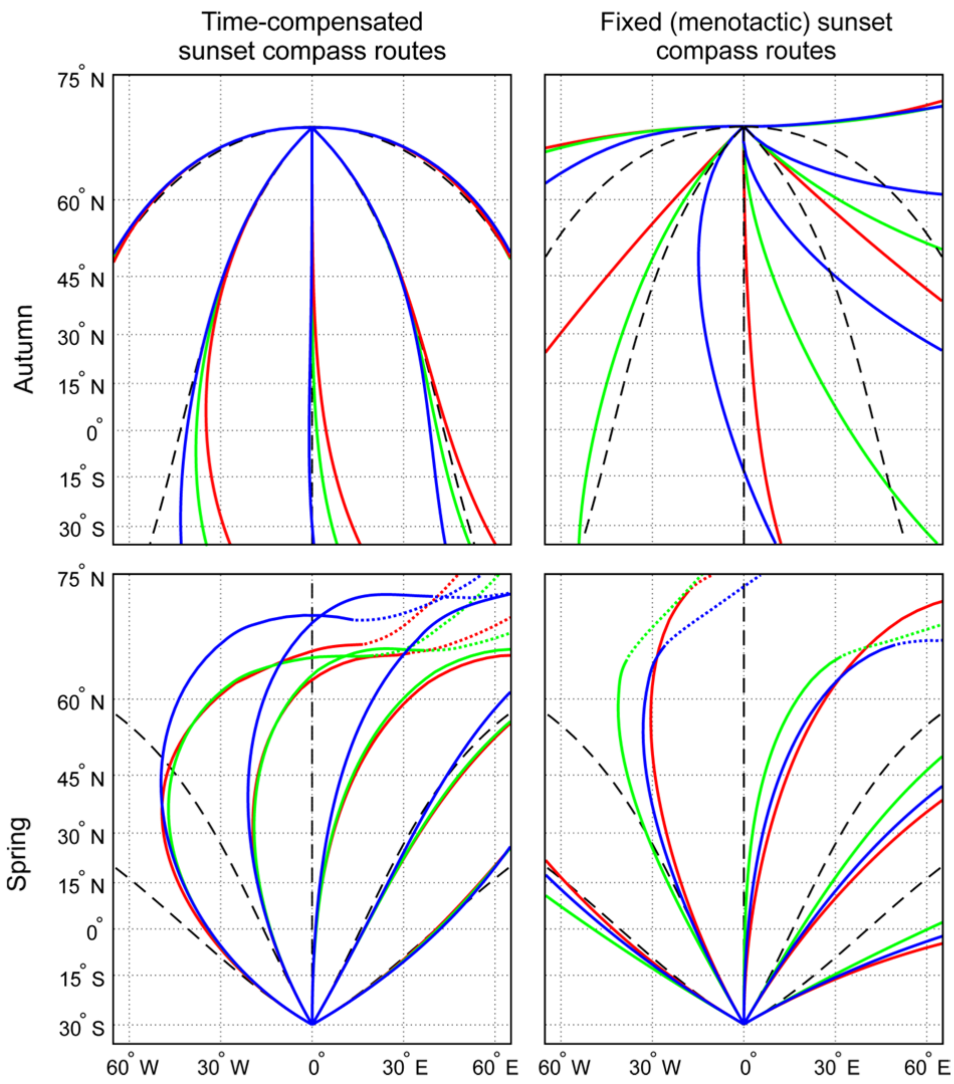

Figure S2. Effect of time of season on flight trajectories of migrants following time-compensated sunset and fixed (menotactic) sunset compass routes. Autumn migration routes were simulated with 1 Aug (blue), 1 Sept (green), and 1 Oct (red) as initial departure dates and with initial departure directions of 90°, 135°, 180°, 225° and 270° from departure locations at latitudes 70°N. Spring migration were simulated with 1 March (blue), 1 April (green), and 1 May (red) as departure dates and with initial departure directions of 300°, 330°, 360°, 30° and 60° from departure locations at latitudes 30°S. All routes were calculated in daily steps of 200 km with a new course for each step based on astronomical conditions at each daily departure location/time and assuming a constant geographic course within a step. Dotted sections of routes indicate situations where the sun did not set anymore once the birds reached higher latitudes, thus where the lowest sun elevation was taken as reference instead. Great circle routes (dark grey dashed) are given for comparison to indicate the shortest routes. The routes are presented in Mercator projection.
