# Supplementary material for: Feasibility of sun and magnetic compass mechanisms in avian long-distance migration
Source: Mov Ecol. 2018 Jun 6;6:8. doi: 10.1186/s40462-018-0126-4 (PMC5989362; doi:10.1186/s40462-018-0126-4)
Supplement: Supplementary file 3 — Figure S3. Time-compensated sunset compass routes during spring migration with initial departure directions of 354°, 356°, 358°, 0°, 2°, 4° and 6°. Spring routes starting at lower latitudes on either side of (or at) the equator are very sensitive to small differences in departure courses due to small differences in sunset directions over latitude and time in the tropics. Great circle routes (dark grey dashed) are given for comparison to indicate the shortest routes. The routes are presented in Mercator projection. (PDF 120 kb) [file 40462_2018_126_MOESM3_ESM.pdf]

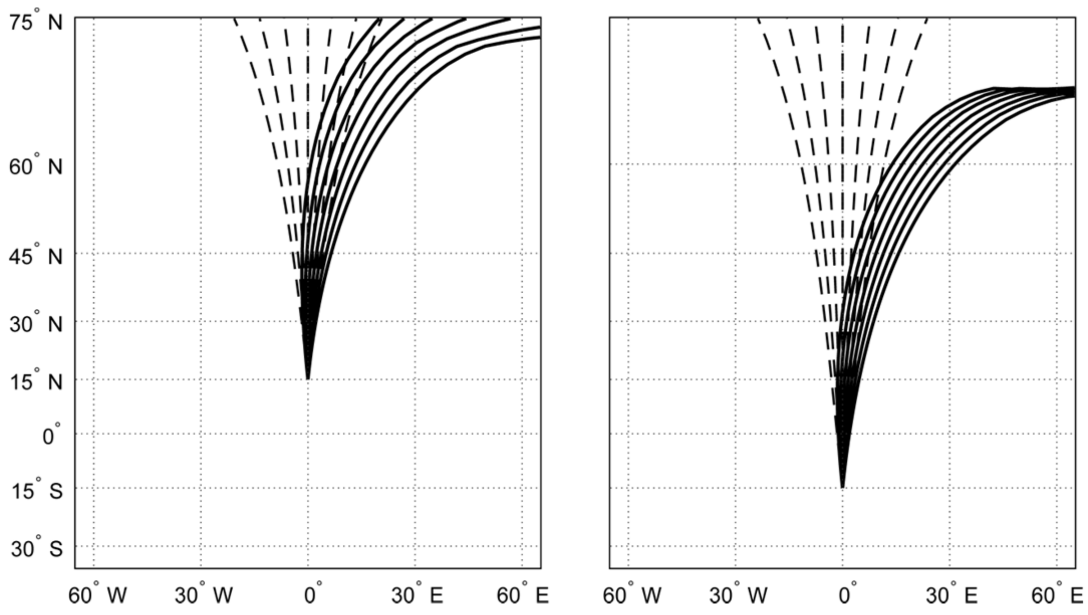

Figure S3. Time-compensated sunset compass routes during spring migration with initial departure directions of 354°, 356°, 358°, 0°, 2°, 4° and 6°. Spring routes starting at lower latitudes on either side of (or at) the equator are very sensitive to small differences in departure courses due to small differences in sunset directions over latitude and time in the tropics. Great circle routes (dark grey dashed) are given for comparison to indicate the shortest routes. The routes are presented in Mercator projection.
