# Supplementary material for: Feasibility of sun and magnetic compass mechanisms in avian long-distance migration
Source: Mov Ecol. 2018 Jun 6;6:8. doi: 10.1186/s40462-018-0126-4 (PMC5989362; doi:10.1186/s40462-018-0126-4)
Supplement: Supplementary file 4 — Figure S4. (A) The time-compensated sunset compass route is deflected near the geographic North Pole because of the rapid changes in absolute directions that the bird is experiencing when flying across longitudes near the poles. Red crosses give the positions of a putative bird departing from Alaska along a time-compensated sunset compass route towards the North Pole, reorienting every 200 km. Gnomonic map projection. (B) Sun position (azimuth) at the equator (0° latitude, 0° longitude) over a 24-h period on spring equinox (21 March). Birds starting near the equator close to spring equinox on a time-compensated sunset compass route will run into problems because of the sudden shift of the sun from the east to the west over the course of about 15 min. See also Additional file 6: Figure S6. (PDF 141 kb) [file 40462_2018_126_MOESM4_ESM.pdf]

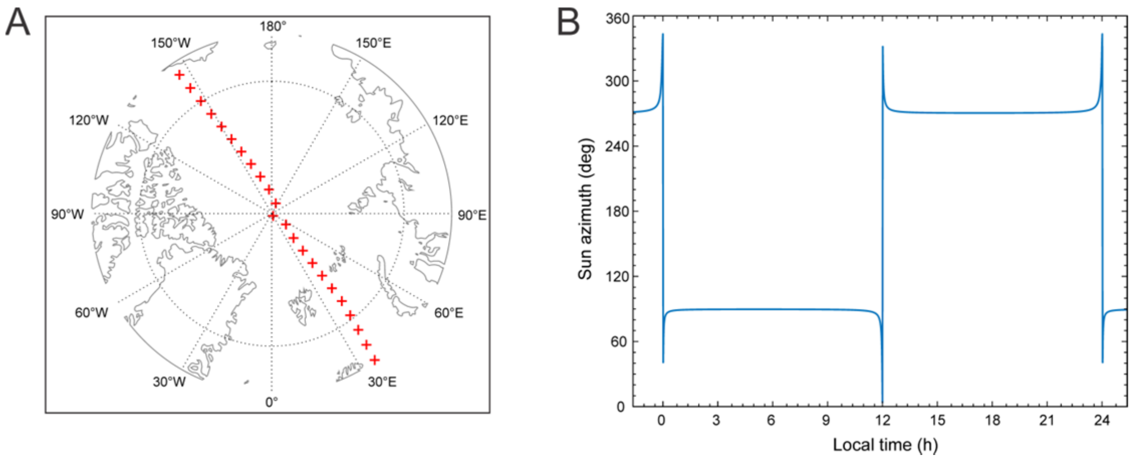

Figure S4. (A) The time-compensated sunset compass route is deflected near the geographic North Pole because of the rapid changes in absolute directions that the bird is experiencing when flying across longitudes near the poles. Red crosses give the positions of a putative bird departing from Alaska along a time-compensated sunset compass route towards the North Pole, reorienting every 200 km. Gnomonic map projection. (B) Sun position (azimuth) at the equator (0° latitude, 0° longitude) over a 24-hour period on spring equinox (21 March). Birds starting near the equator close to spring equinox on a time-compensated sunset compass route will run into problems because of the sudden shift of the sun from the east to the west over the course of about 15 min. See also Additional file 6: Figure S6.
