# Supplementary material for: Feasibility of sun and magnetic compass mechanisms in avian long-distance migration
Source: Mov Ecol. 2018 Jun 6;6:8. doi: 10.1186/s40462-018-0126-4 (PMC5989362; doi:10.1186/s40462-018-0126-4)
Supplement: Supplementary file 5 — Figure S5. Visualisation of the magnetoclinic compass. Magnetoclinic orientation refers to the case where migratory birds fly at a constant “apparent angle of inclination” (γ′ in blue). The apparent angle of inclination is the inclination of the geomagnetic field projected on a plane orthogonal to the bird’s heading or body axis. As inclination changes with latitude, a migrant must change its course in order to keep γ′ constant. In horizontal flight the apparent angle of inclination is a function of the geomagnetic inclination (γ in red) and the bird’s flight course (α in green), according to the relationship tan(γ′) = tan(γ)/ sin(α). The illustration shows the headings of a bird flying along a fixed γ’ in areas with different angles of inclination γ1 (left graph) and γ2 (right graph). The bird maintains a fixed γ′ by adjusting its heading from more westerly directions α1 to more southerly directions α2 with decreasing geomagnetic inclination from γ1 (left graph) and γ2 (right graph). Magnetoclinic orientation will be affected if birds do not fly horizontally and also by wind conditions depending on whether the birds perceive the apparent inclination magnetostatically in relation to their body axis or by a magnetic induction process in relation to their trajectory through the magnetic field, as evaluated by Alerstam (1987: J Exp Biol. 1987;130:63–86). These effects are not included in the simplified geometric explanation in the figure here. (PDF 173 kb) [file 40462_2018_126_MOESM5_ESM.pdf]

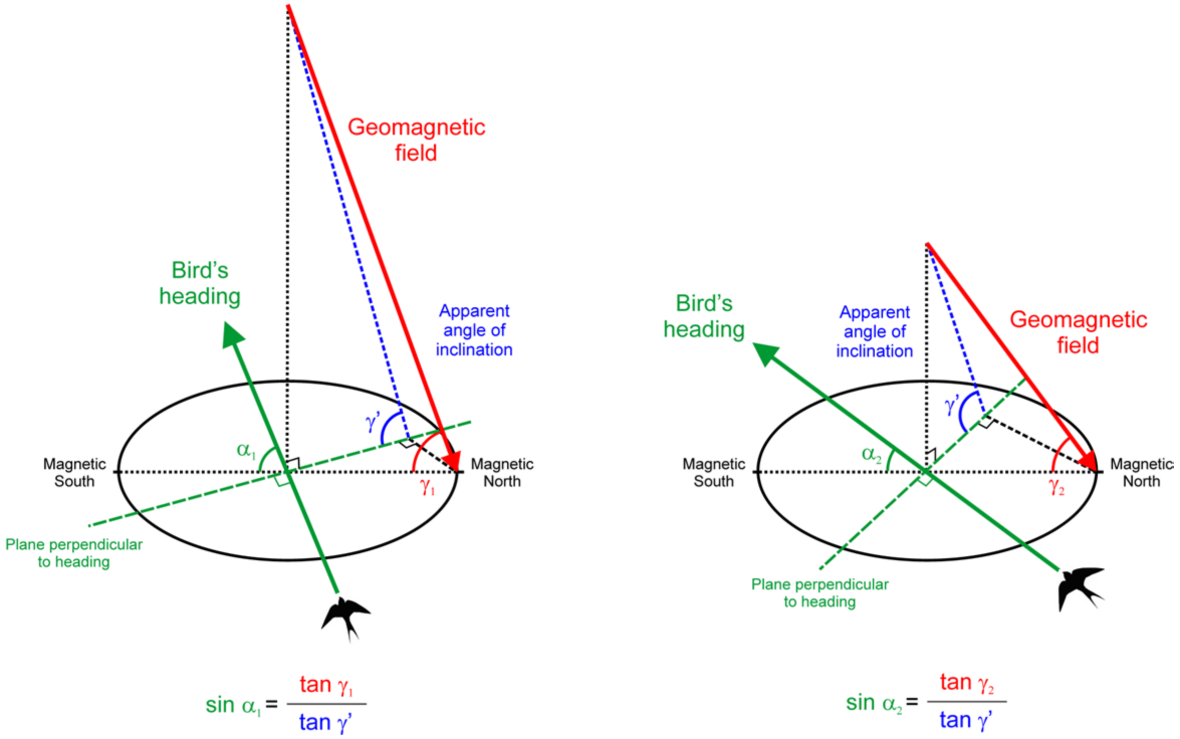

Figure S5. Visualisation of the magnetoclinic compass. Magnetoclinic orientation refers to the case where migratory birds fly at a constant "apparent angle of inclination" (γ´ in blue). The apparent angle of inclination is the inclination of the geomagnetic field projected on a plane orthogonal to the bird's heading or body axis. As inclination changes with latitude, a migrant must change its course in order to keep γ´ constant. In horizontal flight the apparent angle of inclination is a function of the geomagnetic inclination (γ in red) and the bird's flight course (α in green), according to the relationship tan (γ´) = tan (γ)/ sin (α). The illustration shows the headings of a bird flying along a fixed γ´ in areas with different angles of inclination γ_1 (left graph) and γ_2 (right graph). The bird maintains a fixed γ´ by adjusting its heading from more westerly directions α_1 to more southerly directions α_2 with decreasing geomagnetic inclination from γ_1 (left graph) and γ_2 (right graph).

Magnetoclinic orientation will be affected if birds do not fly horizontally and also by wind conditions depending on whether the birds perceive the apparent inclination magnetostatically in relation to their body axis or by a magnetic induction process in relation to their trajectory through the magnetic field, as evaluated by Alerstam (1987: J Exp Biol. 1987;130:63–86). These effects are not included in the simplified geometric explanation in the figure here.
