# Supplementary material for: Feasibility of sun and magnetic compass mechanisms in avian long-distance migration
Source: Mov Ecol. 2018 Jun 6;6:8. doi: 10.1186/s40462-018-0126-4 (PMC5989362; doi:10.1186/s40462-018-0126-4)
Supplement: Supplementary file 6 — Figure S6. Two examples of magnetoclinic compass routes during spring migration starting from the equator (0° latitude; left graph) or 20°S (right graph) with initial departure directions of 354°, 356°, 358°, 0°, 2°, 4° and 6°. Great circle routes (dark grey dashed) are given for comparison to indicate the shortest routes. The routes are presented in Mercator projection. (PDF 171 kb) [file 40462_2018_126_MOESM6_ESM.pdf]

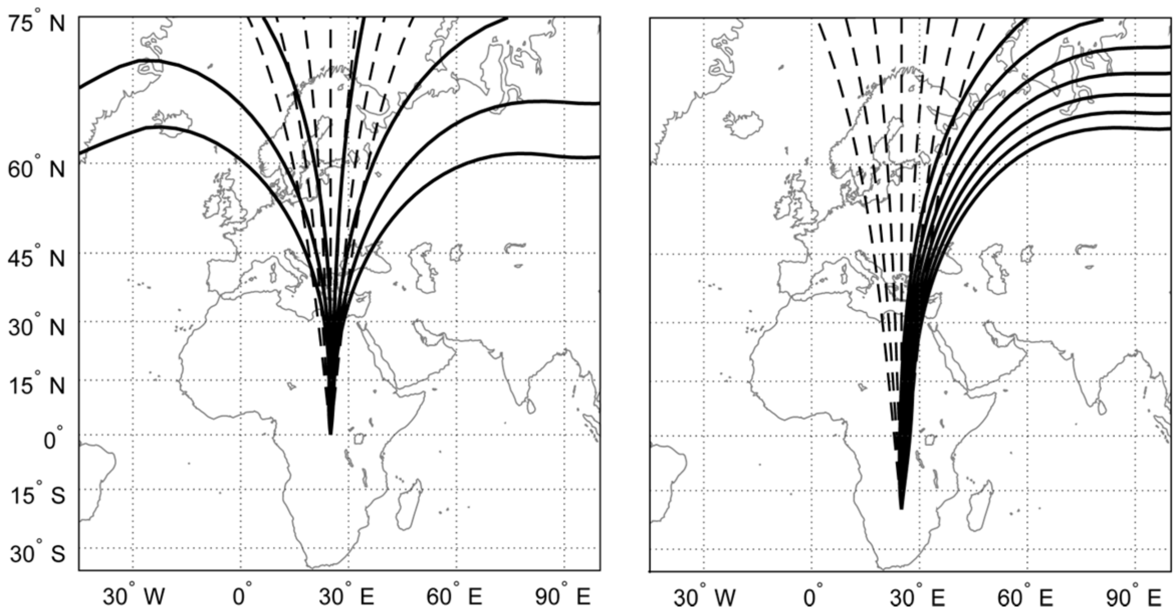

Figure S6. Two examples of magnetoclinic compass routes during spring migration starting from the equator (0° latitude; left graph) or 20°S (right graph) with initial departure directions of 354°, 356°, 358°, 0°, 2°, 4° and 6°. Great circle routes (dark grey dashed) are given for comparison to indicate the shortest routes. The routes are presented in Mercator projection.
