# Supplementary material for: Feasibility of sun and magnetic compass mechanisms in avian long-distance migration
Source: Mov Ecol. 2018 Jun 6;6:8. doi: 10.1186/s40462-018-0126-4 (PMC5989362; doi:10.1186/s40462-018-0126-4)
Supplement: Supplementary file 7 — Figure S7. Explanation for why birds starting from equatorial latitudes during spring migration may not reach their destinations if following a time-compensated sunset compass. Example of a bird departing on spring migration in eastern Africa (12°N, 20°E; blue triangle) on 14 April 2014 towards its destination in Alaska (black dot), advancing 295 km/d. Graphs on the right show the sun ephemeris curves, i.e. the azimuth of the sun relative to Universal time, for three consecutive days illustrated in red, green and turquoise, incl. local sunset (dots in respective colours). The blue ephemeris curves give the azimuth of the sun at the departure location and departure date, which the bird uses as reference. The blue triangles show the sun azimuth at the departure location at the time of local sunset for each of the three days. The bird determines its departure direction at local sunset, but uses the sun ephemeris from the departure location and departure date as reference. Thus, it changes its daily departure direction by the difference between the local sunset azimuth and the azimuth of the sun at that specific time at the departure location and date. The sudden shift in compass direction is the result of the sun changing its position relatively quickly from west to south to east at noon at the departure location. These shifts are most dramatic at the geographic equator, thus affects birds departing from areas close to the equator, and migrate enough days for the local sunset time to coincide with the time of noon at the departure location. Birds can avoid this by updating their inner clock at least once along their journey at higher latitudes and then continue using the sun ephemeris of the reset location as reference for the remaining journey. The map is in Mercator projection. (PDF 236 kb) [file 40462_2018_126_MOESM7_ESM.pdf]

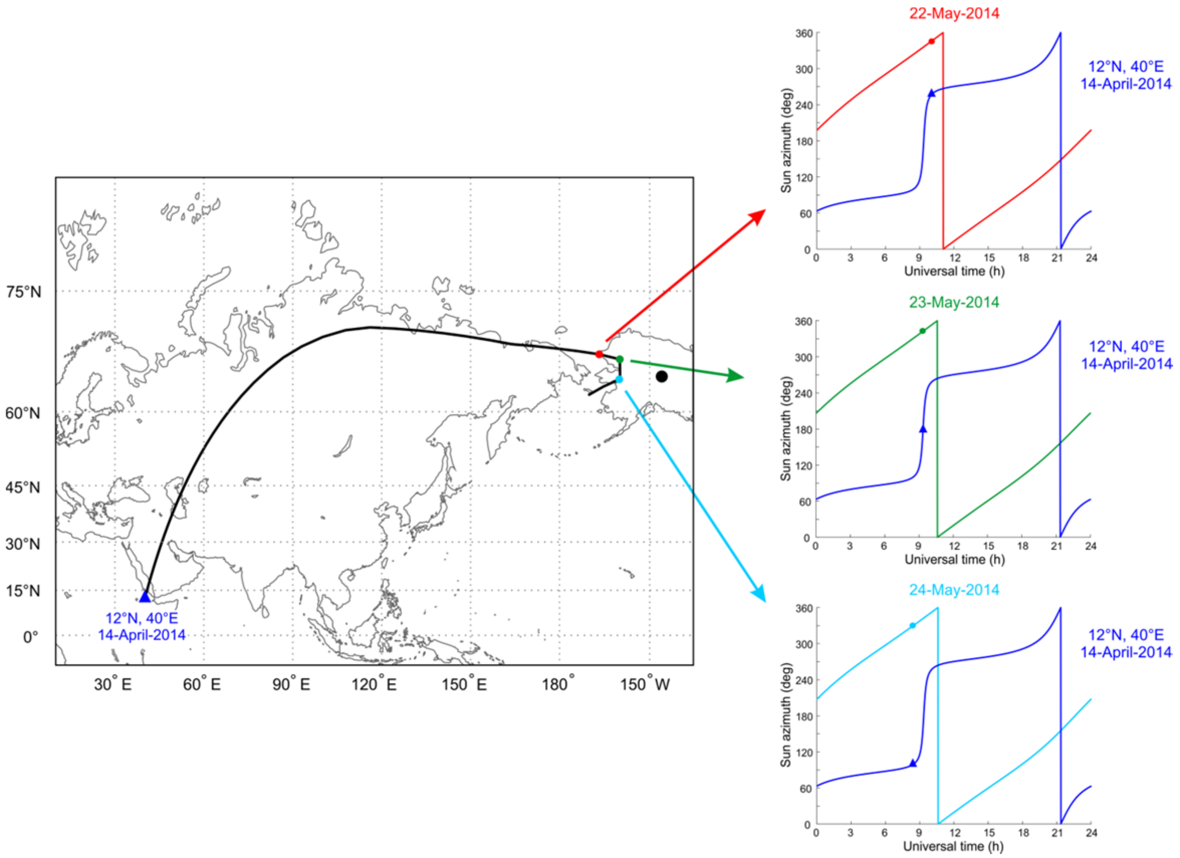

Figure S7. Explanation for why birds starting from equatorial latitudes during spring migration may not reach their breeding areas in Alaska, if following a time-compensated sunset compass. The trajectory on the map illustrates an example of a bird departing on its spring migration in eastern Africa (blue triangle) on 14 April 2014 and advancing 295 km/d. Just before reaching its breeding location in Alaska (black dot), the trajectory of the time-compensated sunset compass makes a sudden change in direction. Graphs on the right show the sun ephemeris curves, i.e. the azimuth of the sun relative to Universal time, for three consecutive days illustrated in different colours (red, green and turquoise). The dots in the respective colours indicate local sunset. The blue ephemeris curves give the azimuth of the sun at the departure location (12°N, 20°E) at the departure date (14 April 2014), which birds migrating with a time-compensated sunset compass use as reference as long as they do not update their inner clock. The blue triangles show the sun azimuth at the departure location at the time of local sunset (indicated by the red, green and turquoise dot) for each of the three days. Birds migrating with a time-compensated sunset compass determine their departure direction at local sunset, but use the sun ephemeris from the departure location and departure date as reference. Thus, they change their daily departure direction by the difference between the local sunset azimuth and the azimuth of the sun at that specific time at the departure location and departure date. The sudden shift in compass direction is the result of the sun changing its position relatively quickly from west to south to east at noon at the departure location. These shifts are most dramatic at the geographic equator, thus affects birds departing from areas close to the equator, and migrate long enough (measured in days) for the local sunset time to coincide with the time of noon at the departure location. Birds can avoid such a scenario by updating their inner clock at least once along their journey at higher latitudes and then continue using the sun ephemeris of the reset location as reference for the remaining journey. The map is in Mercator projection.
