# Supplementary material for: Feasibility of sun and magnetic compass mechanisms in avian long-distance migration
Source: Mov Ecol. 2018 Jun 6;6:8. doi: 10.1186/s40462-018-0126-4 (PMC5989362; doi:10.1186/s40462-018-0126-4)
Supplement: Supplementary file 8 — Figure S8. (A) Magnetoclinic compass routes of a northern wheatear (B070) departing from 66°N at different longitudes (155° E, 160° E, 175° W, 160° W, 155° W; black triangles) in westerly directions (270° relative to magnetic North). Because of the different angles of magnetic inclination at the different starting locations (γ = 79.1°, 76.9°, 75.5°, 75.2°, 76.2° from easterly to westerly sites), the bird starts with different apparent angles of inclination (γ′ = γ). Depending on the distribution of magnetic inclination, the birds are either led immediately southwards (solid lines, where γ′ > γ) or along the magnetic inclination isoclines (dashed lines, where γ > γ′). (B) Magnetoclinic compass routes of the same bird starting from its initial departure location with different γ′. It is possible for the bird to reach its destination (black dot at 13°N, 37°E) by using a magnetoclinic compass and without resetting the compass along the journey, but the path is highly sensitive to minute changes of the apparent angle of inclination (sensitivity < 2 × 10− 8 deg.), making this strategy highly unlikely. The maps are in Mercator projection. (PDF 388 kb) [file 40462_2018_126_MOESM8_ESM.pdf]

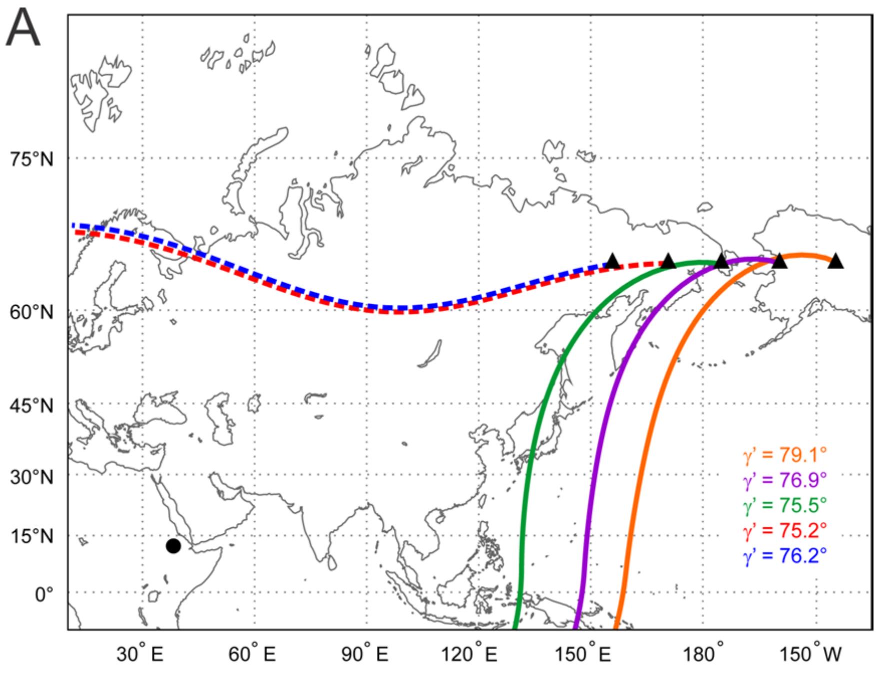

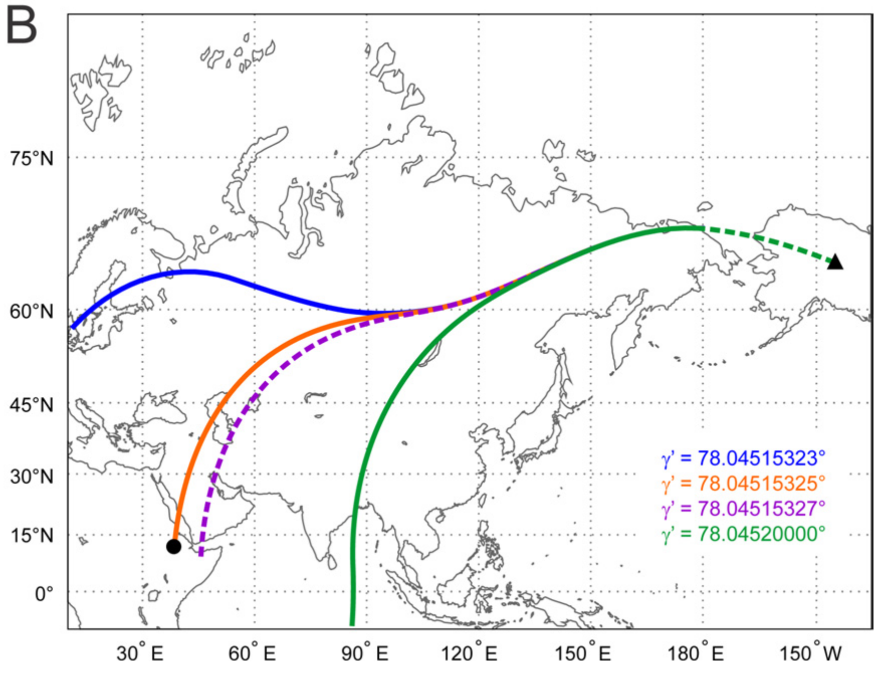

Figure S8. (A) Magnetoclinic compass routes of a northern wheatear (B070) departing from 66°N at different longitudes (155° E, 160° E, 175° W, 160° W, 155° W; black triangles) in westerly directions (270° relative to magnetic North). Because of the different angles of magnetic inclination at the different starting locations (γ = 79.1°, 76.9°, 75.5°, 75.2°, 76.2° from easterly to westerly sites), the bird starts with different apparent angles of inclination ($\gamma' = \gamma$). Depending on the distribution of magnetic inclination, the birds are either led immediately southwards (solid lines, where $\gamma' > \gamma$) or along the magnetic inclination isoclines (dashed lines, where $\gamma > \gamma'$). (B) Magnetoclinic compass routes of the same bird starting from its initial departure location with different γ'. It is possible for the bird to reach its destination (black dot at 13°N, 37°E) by using a magnetoclinic compass and without resetting the compass along the journey, but the path is highly sensitive to minute changes of the apparent angle of inclination (sensitivity < 2×10^{-8} deg.), making this strategy highly unlikely. The maps are in Mercator projection.
